# Biogenesis of *C*-Glycosyl Flavones and Profiling of Flavonoid Glycosides in Lotus (*Nelumbo nucifera*)

**DOI:** 10.1371/journal.pone.0108860

**Published:** 2014-10-03

**Authors:** Shan-Shan Li, Jie Wu, Li-Guang Chen, Hui Du, Yan-Jun Xu, Li-Jing Wang, Hui-Jin Zhang, Xu-Chen Zheng, Liang-Sheng Wang

**Affiliations:** 1 Key Laboratory of Plant Resources and Beijing Botanical Garden, Institute of Botany, Chinese Academy of Sciences, Beijing, China; 2 University of Chinese Academy of Sciences, Beijing, China; 3 Institute of Forest Protection, Chinese Academy of Forestry, Beijing, China; 4 College of Science, China Agricultural University, Beijing, China; 5 College of Horticulture, Nanjing Agricultural University, Nanjing, China; Bangor University, United Kingdom

## Abstract

Flavonoids in nine tissues of *Nelumbo nucifera* Gaertner were identified and quantified by high-performance liquid chromatography with diode array detector (HPLC-DAD) and HPLC-electrospray ionization-mass spectrometry (HPLC-ESI-MS^n^). Thirty-eight flavonoids were identified; eleven *C*-glycosides and five *O*-glycosides were discovered for the first time in *N. nucifera*. Most importantly, the *C-*glycosyl apigenin or luteolin detected in lotus plumules proved valuable for deep elucidation of flavonoid composition in lotus tissues and for further utilization as functional tea and medicine materials. Lotus leaves possessed the significantly highest amount of flavonoids (2.06E3±0.08 mg 100 g^−1^ FW) and separating and purifying the bioactive compound, quercetin 3-*O*-glucuronide, from leaves showed great potential. In contrast, flavonoids in flower stalks, seed coats and kernels were extremely low. Simultaneously, the optimal picking time was confirmed by comparing the compound contents in five developmental phases. Finally, we proposed the putative flavonoid biosynthesis pathway in *N. nucifera*.

## Introduction

Component analysis of bioactive compounds including flavonoids, proanthocyanins and essential oils, has been the target of much research [1]–[3]. Among these, flavonoids are the prevalent phytochemicals highlighted in recent research because of their diverse biological activities, including antioxidant, anti-inflammatory, antiallergic, antimutagenic, antibiotic and anticarcinogenic properties [4]. Additionally, flavonoids have also gained the attention of many researchers as chemotaxonomic markers [5].

Flavonoids, with a C6-C3-C6 skeleton, are biosynthesized by a series of condensation reactions between hydroxycinnamic acid (B ring) and malonyl residues (A ring). As a result, these compounds exhibit a two-absorption band structure: a cinnamoyl system at 300–400 nm (Band I) and a benzoyl system at 240–280 nm (Band II). In plants, flavonoids are found in various modified forms, generated by hydroxylation, methylation, acylation and glycosylation, among which glycosylated flavonoids are by far the most common natural compounds. In addition, glycosylation may occur as *C*-glycosyl flavonoids with formation of a C-C bond by direct linkage of the sugar to the basic skeleton of the flavonoid. So far, *C*-glycosylation has been found only at the C6 and/or C8 position.

*Nelumbo nucifera* Gaertner, considered to be a traditional ornamental and medicinal plant, has been cultivated all over China. It is usually used as a vegetable or in Chinese traditional herbal medicines, and each part of the lotus is valuable. For example, lotus seed kernels, rhizomes, young leaves and flower petals are usually used as raw food materials. Lotus leaves and plumules have been highly valued in traditional Chinese medicine. Moreover, petals and stamens, rich in bioactive components such as flavonoids and alkaloids, are used in the treatment of tissue inflammation, cancer, skin diseases and for use as antidotes [6].

Weishan Lake, the fifth biggest fresh water lake in China, possesses more than thirty thousand hectares of *N. nucifera* cultivation. According to preliminary statistics, it can produce approximately two thousand kilograms of dried seed kernels and ten thousand kilograms of dessicated lotus leaves each year [7]. The remaining tissues of lotus are mostly wasted every year. Therefore, the comprehensive development of uses for various lotus tissues is believed to be a promising avenue of inquiry. This study profiled the flavonoids in various tissues in lotus, and plentiful *C-*glycosyl flavones were detected in lotus plumules for the first time. Therefore, the application of lotus plumules in medicine is intriguing and likely to be the next frontier.

## Results and Discussion

### 1. Method validation

To verify the reliability of the optimized separation method, the analytical parameters were determined for anthocyanins with cyanidin 3-*O*-β-D-glucopyranoside (Cy-3-Glc) and other flavonoids with quercetin 3-*O*-α-L-rhamnopyranosyl-(1→6)-β-D-glucopyranoside (rutin). Table S.1 shows the results of method validation for two external standards (Cy-3-Glc, rutin). The calibration curves were established with five concentrations of each standard in triplicate. Results showed good linearity in relatively wide concentration ranges for both standards at 525 and 350 nm (r^2^≥0.9993). The limits of detection (LODs) and the limits of quantitation (LOQs) were separately defined as a signal-to-noise ratio of 3∶1 and 10∶1. The LOD and LOQ were obtained for Cy-3-Glc (0.51 and 1.68 µg mL^−1^) and rutin (S I, 0.28 and 0.94 µg mL^−1^; S II, 0.68 and 2.27 µg mL^−1^) (Table S.1).

The precision of the optimized method was studied by examining the repeatability (intra-day analysis, n = 6) and intermediate precision (inter-day analysis, n = 3) for all the compounds separated from petals, seed coats and plumules because these three tissues contained all the anthocyanins and flavonoid components. Six samples of these tissues were extracted and analyzed on the same day to determine the intra-day precision. Three samples per day were also extracted and evaluated on three consecutive days to determine the inter-day precision. The results showed that the relative standard deviations (RSDs) of the 38 compounds were less than 2.51% in inter-day test and less than 2.35% for intra-day analysis (Table S.2). The low RSD values obtained for all compounds demonstrated the high repeatability and intermediate precision of the methods proposed here.

### 2. Identification of flavonoids

#### 2.1 Qualitative analysis for anthocyanins

Compared with the qualitative analysis of the other flavonoids in lotus tissues, the identification of anthocyanin glycosides was straightforward. According to published MS spectra [8], five peaks were readily identified as delphinidin 3-*O*-glucoside (Dp-3-Glc, a1), cyanidin 3-*O*-glucoside (Cy-3-Glc, a2), petunidin 3-*O*-glucoside (Pt-3-Glc, a3), peonidin 3-*O*-glucoside (Pn-3-Glc, a4) and malvidin 3-*O*-glucoside (Mv-3-Glc, a5). Table S.3 shows the characteristics of anthocyanins separated from lotus petals.

#### 2.2 Identification of *O*-flavonoids

**Figure 1:**
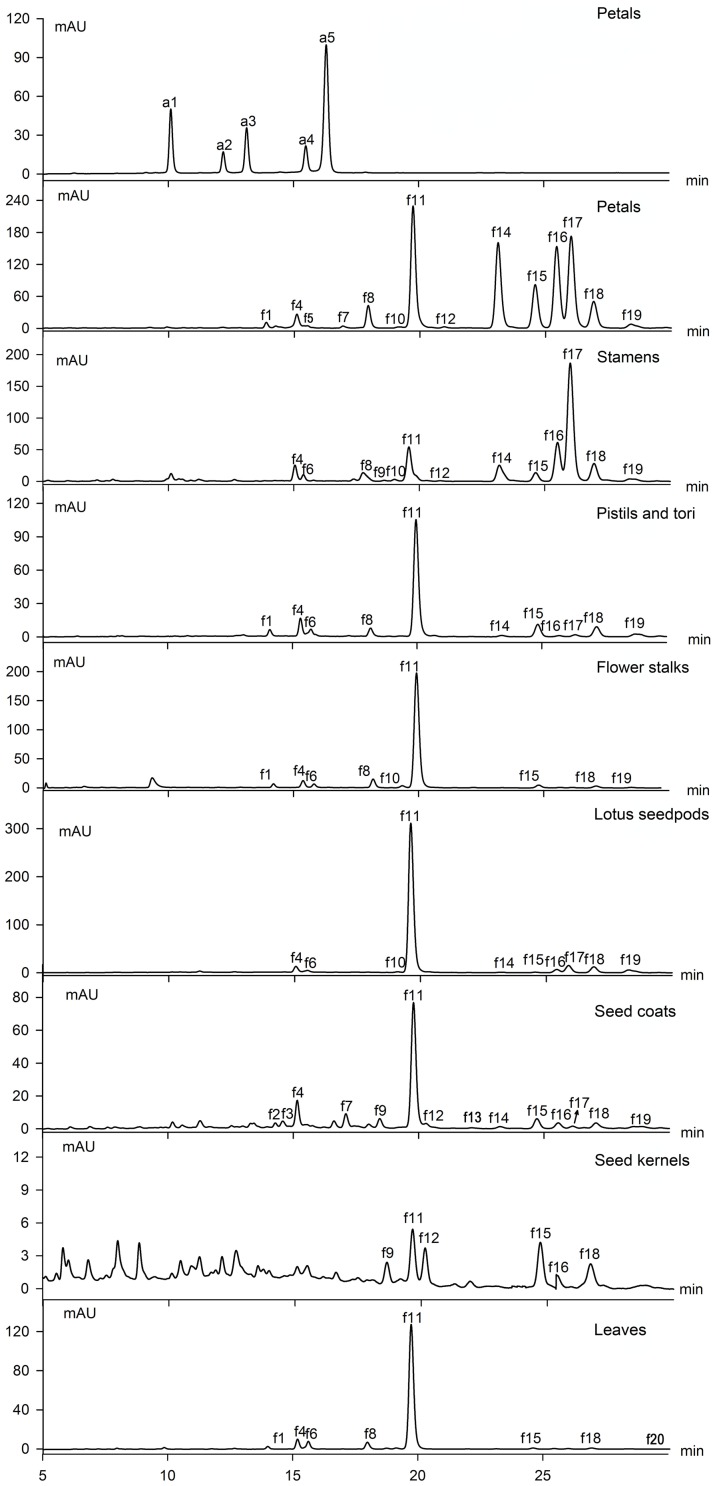
HPLC elution profiles of flavonoids from different tissues in lotus.

**Figure 2:**
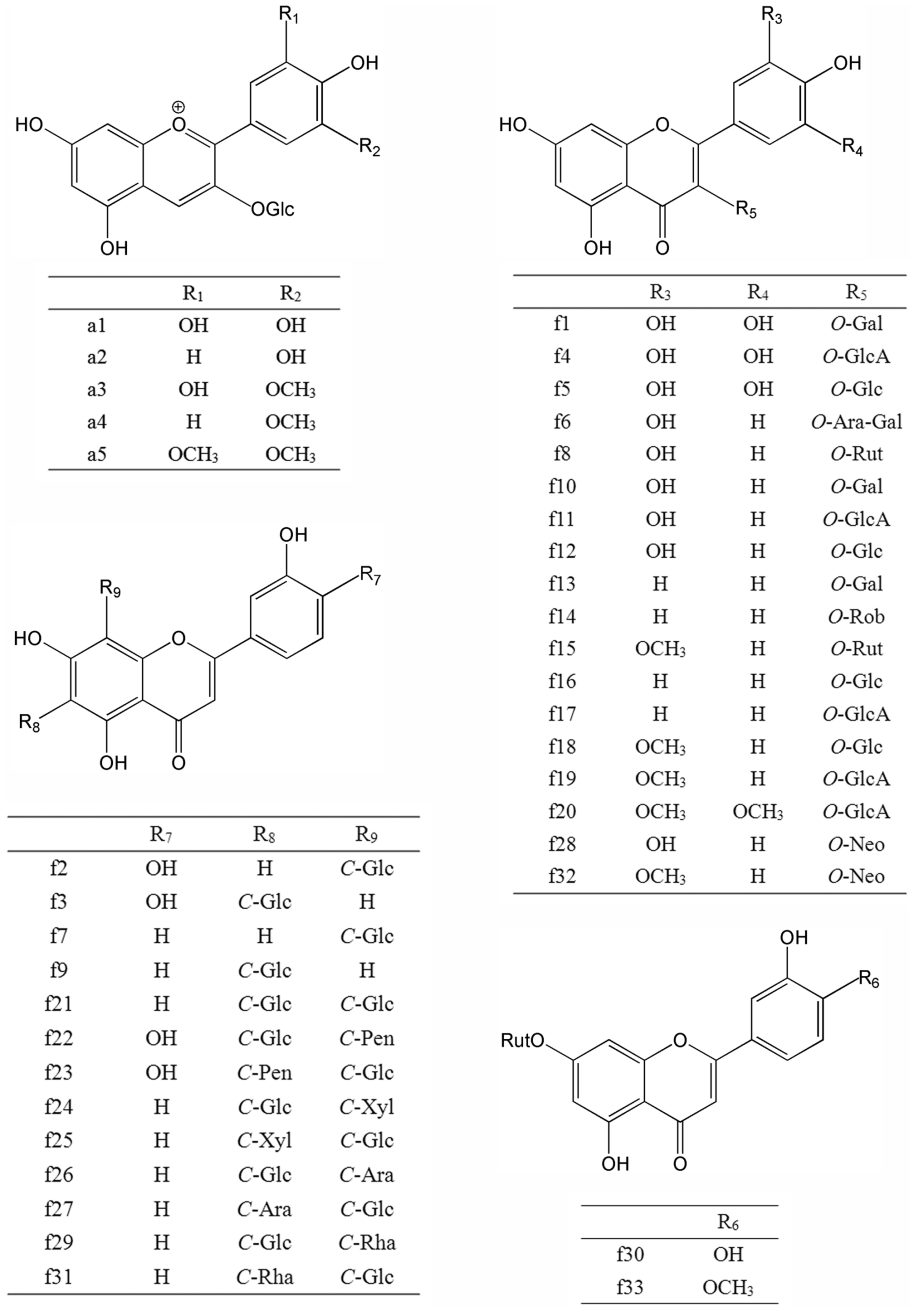
The chemical structure scheme of flavonoids detected in lotus tissues.

In this study, 18 *O-*flavonols and two *O-*flavones have been detected by the characterization of UV-vis absorption spectroscopy (Fig. 1, 2). The aglycone ions at *m*/*z* 319 [Y_0_]^+^ in positive ion (PI) mode and *m*/*z* 317 [Y_0_]^−^ in negative ion (NI) mode or the radical aglycone ion at *m*/*z* 316 [Y_0_−H]^−^ of peak f1, f4 and f5 indicated that they were myricetin monosaccharides (Table 1). According to Mabry et al., glycosylation of the 3-, 5- or 4′-hydroxyl group produces a 12–17, 5–15 or 3–10 nm hypsochromic shift in Band I (in methanol), respectively. Substitution of the hydroxyl groups at positions other than 3, 5, and 4′ has little or no effect on the UV spectrum. Obviously, compounds f1, f4 and f5 showed 16.7–20.1 nm hypsochromic shifts in Band I in 70% methanol aqueous solution and they were all assigned as 3-*O*-glycosides [9]. Glycosides with galactose eluted before their corresponding glucosides [10]. Thus, f1 and f5 were identified as myricetin 3-*O*-galactoside (My-3-Gal) and myricetin 3-*O*-glucoside (My-3-Glc), respectively [11], [12]. The MS fragmentation data (loss of 176 u) of peak f4 suggested a glucuronide substituent. Thus, it was assigned as myricetin 3-*O*-glucuronide (My-3-GlcA) [11]. Similarly, with the deprotonated aglycone ion at *m*/*z* 345[Y_0_]^−^ and a fragment ion of 176 u, peak f20 was tentatively identified as syringetin 3-glucuronide (Sy-3-GlcA) considering the UV-vis spectrum [13].

**Table 1:** UV-Vis absorption maxima and main ESI-MS^n^ peaks of *O*-glycoside flavonoids separated from *N. nucifera*.

No	Rt (min)^a^	UV λ_max_ (nm)	ESI-NI (*m/z*)	ESI-PI (*m/z*)	Identification^b^	Ref
f1	13.63	257.5, 354.4	479[M−H]^−^, 316[Y_0_−H]^•−^	503[M+Na]^+^, 481[M+H]^+^, 319[Y_0_]^+^	My-3-Gal	Chen et al., 2012a
f4	15.04	261.6, 357.3	493[M−H]^−^, 317[Y_0_]^−^	517[M+Na]^+^, 495[M+H]^+^, 319[Y_0_]^+^	My-3-GlcA	Chen et al., 2012a
f5	15.13	262.1, 353.9	479[M−H]^−^, 316[Y_0_−H]^•−^	503[M+Na]^+^, 319[Y_0_]^+^	My-3-Glc	Huang et al., 2010
f6	15.32	257.0, 354.5	595[M−H]^−^, 300[Y_0_−H]^•−^	619[M+Na]^+^, 303[Y_0_]^+^	Qu-3-Ara-Gal	Jung et al., 2008
f8	17.60	257.3, 354.6	609[M−H]^−^, 301[Y_0_]^−^	633[M+Na]^+^, 611[M+H]^+^, 303[Y_0_]^+^	Rutin	Standard
f10	18.97	257.1, 354.3	463[M−H]^−^, 300[Y_0_−H]^•−^	487[M+Na]^+^, 303[Y_0_]^+^	Hyperoside	Standard
f11	19.58	257.3, 353.7	477[M−H]^−^, 301[Y_0_]^−^	501[M+Na]^+^, 479[M+H]^+^, 303[Y_0_]^+^	Qu-3-GlcA	Goo et al., 2009
f12	20.27	254.1, 355.3	463[M−H]^−^, 300[Y_0_−H]^•−^	487[M+Na]^+^, 303[Y_0_]^+^	Isoquercitrin	Standard
f13	21.83	257.7, 350.1	447[M−H]^−^, 284[Y_0_−H]^•−^	471[M+Na]^+^, 287[Y_0_]^+^	Ka-3-Gal	Yang et al., 2009
f14	23.21	266.3, 346.8	593[M−H]^−^, 284[Y_0_]^−^	617[M+Na]^+^, 449[M+H−146]^+^, 287[Y_0_]^+^	Ka-3-Rob	Yang et al., 2009
f15	24.13	255.8, 354.6	623[M−H]^−^, 461Y_0_^1^]^−^, 315[Y_0_]^−^	647[M+Na]^+^, 479[M+H−146]^+^, 317[Y_0_]^+^	Is-3-Rut	Lim et al., 2006
f16	25.38	266.1, 347.3	447[M−H]^−^, 284[Y_0_−H]^•−^	471[M+Na]^+^, 287[Y_0_]^+^	Ka-3-Glc	Standard
f17	25.54	269.2, 345.9	461[M−H]^−^, 285[Y_0_]^−^	485[M+Na]^+^, 287[Y_0_]^+^	Ka-3-GlcA	Yang et al., 2009
f18	26.44	255.2, 356.2	477[M−H]^−^, 314[Y_0_−H]^•−^	501[M+Na]^+^, 317[Y_0_]^+^	Is-3-Glc	Standard
f19	27.91	256.4, 353.9	491[M−H]^−^, 315[Y_0_]^−^	515[M+Na]^+^, 317[Y_0_]^+^	Is-3-GlcA	Chen et al., 2012a
f20	28.12	254.0, 355.8	521[M−H]^−^, 345[Y0]^−^	545[M+Na]^+^, 523[M+H]^+^, 347[Y_0_]^+^	Sy-3-GlcA	Noelia et al., 2009
f28	38.03	256.1, 354.9	609[M−H]^−^, 463[M−H−146]^−^, 301[Y_0_]^−^, 300[Y_0_−H]^•−^	303[Y_0_]^+^, 487[M+Na−146]^+^, 633[M+Na]^+^	Qu-3-Neo	Buttery et al., 1975
f30	44.46	255.2, 348.0	593[M−H]^−^, 447[M−H−146]^−^, 285[Y_0_]^−^, 284[Y_0_−H]^•−^	617[M+Na]^+^, 471[M+Na−146]^+^, 287[Y_0_]^+^	Lu-7-Rut	Lin et al., 2010
f32	56.68	251.3, 353.9	623[M−H]^−^, 477[M−H−146]^−^, 315[Y_0_]^−^, 314[Y_0_−H]^•−^	647[M+Na]^+^, 317[Y_0_]^+^	Is-3-Neo	Tao et al., 2011
f33	63.54	253.2, 346.3	607[M−H]^−^, 299[Y_0_]^−^	631[M+Na]^+^, 301[Y_0_]^+^	Di-7-Rut	Lin et al., 2010

a Rt: retention time on HPLC analysis.

b My: myricetin; Qu: quercetin; Ka: kaempferol; Is: isorhamnetin; Sy: Syringetin; Lu: luteolin; Di: Diosmetin; Gal: galactose; GlcA: glucuronic acid; Glc: glucoside; Ara: arabinose; Rut: rutinose; Rob: robinobiose; Neo: neohesperidose.

The following five major peaks (f6, f8, f10, f11, f12) were all identified as quercetin derivatives with the aglycone fragment ions at *m*/*z* 303 [Y_0_]^+^ in positive-ion (PI) mode and the radical aglycone ion at *m*/*z* 300 [Y_0_−H]^−^ or the aglycone ion at *m*/*z* 301 [Y_0_]^−^ in negative-ion (NI) mode. Peaks f8, f10, f12 co-eluting with their standards were identified as quercetin 3-*O*-α-L-rhamnopyranosyl-(1→6)-β-D-glucopyranoside (rutin), quercetin 3-*O*-β-D-galactopyranoside (hyperoside) and quercetin 3-*O*-β-D-glucopyranoside (isoquercitrin), which accorded with previous reports in lotus petals and leaves [8], [14]. Generally, the fragment ions of diglycoside vary in NI mode depending on the linkage between the two monosaccharides. The radical aglycone ion [Y_0_−H]^−^ is prone to be generated in the case of a 1,2-linkage between the two monosaccharide, while the [Y_0_]^−^ ion is indicative of a 1,6-linkage [15], [16]. Therefore, the higher abundance of the radical aglycone is indicative of a 1,2-linkage. Integrating the precursor ion at *m*/*z* 595 [M−H]^−^ and UV-vis spectrum, peak f6 was assigned as quercetin 3-*O*-arabinopyranosyl-(1→2)-galactopyranoside (Qu-3-Ara-Gal) [17]. Peak f11 showed similar mass spectrometric behaviors with peak 4, and was identified as quercetin 3-*O*-glucuronide (Qu-3-GlcA), which has been reported as the dominant flavonoid in lotus leaves [14].

Combined with their elution order and UV-vis spectrum, peaks f13, f14, f16 and f17 producing major fragments at *m*/*z* 287 [Y_0_]^+^ in PI mode and *m*/*z* 284 [Y_0_−H]^−^ or 285 [Y_0_]^−^ in NI mode were identified as kaempferol glycosides. Among them, f16 was confirmed as kaempferol 3-*O*-β-D-glucopyranoside (Ka-3-Glc) by co-elution with the corresponding standard, while f13, f14 and f17 were separately assigned as kaempferol 3-*O*-galactoside (Ka-3-Gal), kaempferol 3-*O*-robinobioside (Ka-3-Rob), kaempferol 3-*O*-glucuronide (Ka-3-GlcA) by referring to the flavonoids reported in lotus flower petals [8].

With the ions at *m*/*z* 623 [M-H]^−^ and *m*/*z* 315 [Y_0_]^−^ (loss of 308 mass unit) and similar mass spectrometric behaviors with peak f8, peak f15 was identified as isorhamnetin 3-*O*-rutinoside (Iso-3-Rut), which was reported in lotus stamens [18]. Peak f18 was isorhamnetin 3-*O*-β-D-glucopyranoside (Iso-3-Glc), confirmed by co-elution with the standard. The MS fragmentation data (loss of 176 u) of peak f19 suggested that it was isorhamnetin 3-*O*-glucuronide (Iso-3-GlcA) [11].

The fragment ions of f30 and f33 were similar with f8 and f15, suggesting that both possessed a rutinoside substitution. Compound f33 was identified as the diosmetin plus a rutinose with the ions at *m*/*z* 607 [M−H]^−^ and *m*/*z* 299 [Y_0_]^−^. Peak f30 produced pronounced molecular ion at *m*/*z* 593 [M−H]^−^ and aglycone ion at *m*/*z* 285 [Y_0_]^−^ in NI mode, and 617 [M+Na]^+^ and 287 [Y_0_]^+^ in PI mode, which were the features of kaempferol or luteolin plus a rutinose. The aglycone was subsequently confirmed as luteolin, based on the UV λ_max_ (Band II) at 255.2 nm [19]. With the ions at *m*/*z* 609 [M−H]^−^ and *m*/*z* 300 [Y_0_]^−^ of f28 and *m*/*z* 623 [M−H]^−^ and *m*/*z* 314 [Y_0_]^−^ of f32, f28 and f32 were assigned as quercetin and isorhamnetin derivatives, respectively. The interglycosidic linkage in f28 and f32 was C1→C2 due to the relatively higher abundance of the radical aglycone at *m/z* 300 and *m/z* 314 [Y_0_−H]− to the aglycone product ion at *m/z* 301 and *m/z* 315 [Y_0_]^−^ [15]. As expected, the characteristic of UV absorbance wavelength differed notably with different glycosylation positions. For example, the Band I of flavonoid 7-*O*-glycoside caused bathochromic shifts compared with 3-*O*-glycoside [19]. By comparing the UV absorbance wavelength of Band I with earlier study in chrysanthemum flower, soybean and the pollen of *Typha angustifolia*, compounds f28, f30, f32 and f33 were assigned as quercetin 3-*O*-neohesperidoside (Qu-3-Neo), luteolin 7-*O*-rutinoside (Lu-7-Rut), isorhamnetin 3-*O*-neohesperidoside (Is-3-Neo) and diosmetin 7-*O*-rutinoside (Di-7-Rut), respectively [20]–[22]. They were all reported in *N. nucifera* for the first time.

#### 2.3 Identification of *C*-flavones

**Figure 3:**
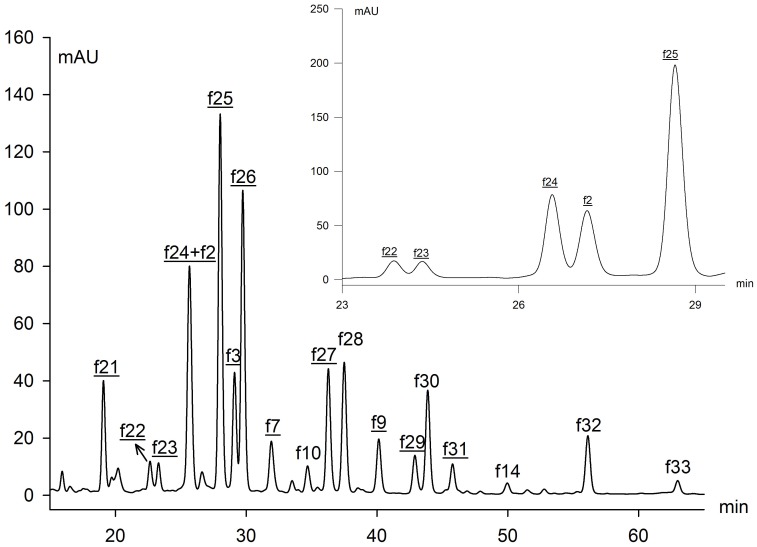
HPLC elution profiles of flavonoids in lotus plumules. The peak numbers of *C*-glycosides were underlined and corresponded to those in Table 1, 2. Peak (f24+f2) achieved a proper separation with another elution gradient.

It was more challenging to identify the *C-*glycosides in lotus plumules due to a lack of previous reports (Fig. 2, 3). From UV spectra, resistance to acidic hydrolysis and a series of fragments [(M−H)−60]^−^, [(M−H)−90]^−^ and [(M−H)−120]^−^, it was deduced that a number of *C-*glycosyl flavones were present in lotus plumules. Abad-Garcia et al. indicated that the spectrum can be differentiated between apigenin (Band I: 336–338 nm) and luteolin (Band I: 342–349 nm) glucosides [23]. Integrating the deprotonated molecule ions at *m*/*z* 447 or 431 [M−H]^−^ and the protonated ions at *m*/*z* 449 [M+H]^+^ or the sodium adduct at *m*/*z* 455 [M+Na]^+^ in both NI and PI modes, peaks f2 and f3 were tentatively characterized as luteolin hexosides, while f7 and f9 were assigned as apigenin hexosides. Meanwhile, the mass spectra data suggested f2 and f7 were isomers of f3 and f9, respectively. Specifically, the [(M+H)−4H_2_O]^+^ ion, characteristic of *C-*6-isomers, was of higher abundance in compounds f3 and f9, whereas the diagnostic ion [^0,3^X]^+^ for *C-*8-hexosides was more abundant in peaks f2 and f7 [23]. Finally, peaks f2, f3, f7and f9 were separately identified as luteolin 8-*C*-β-D-glucopyranoside (orientin), luteolin 6-*C*-β-D-glucopyranoside (isoorientin), apigenin 8-*C*-β-D-glucopyranoside (vitexin) and apigenin 6-*C*-β-D-glucopyranoside (isovitexin), and they were further confirmed by co-elution with their corresponding standards. The results coincided with the previous reports that *C-*8-glycoside eluted before its *C-*6-isomer [10], [24]. Orientin and isoorientin were previously separated from lotus embryo [25].

Based on molecular ions, fragment ions and the diagnostic ions [^0,2^X−H_2_O]^+^ in PI mode, the other *C-*glycosides in the lotus plumule were di-*C*-glycosides [23]. According to Ferreres et al., the ions of the aglycone plus the residues of the sugar, i.e. A+84/83 and A+113, indicated the type of the aglycone, and hence two kinds of aglycone (apigenin: f21, f24, f25, f26, f27, f29, f31; luteolin: f22, f23) were obtained [24]. The fragment ions of peak f21 at *m*/*z* 593[M−H]^−^, 383[A+113] and 353[A+83] suggested the presence of di-*C*-hexosyl apigenin. It was finally assigned as apigenin di-*C*-glucoside.

Peaks f24, f25, f26 and f27, showing the same fragment ions at *m*/*z* 563[M−H]^−^, 503[(M−H)−60]^−^, 473[(M−H)−90]^−^, 443[(M−H)−120]^−^, 383[A+113] and 353[A+83], were isomers, namely 6-*C*-pentosyl-8-*C*-glucosyl apigenin or 6-*C*-glucosyl-8-*C*-pentosyl apigenin. Since *C*-6-isomers were easier to fragment than the *C*-8-isomers, the ion [(M−H)−60]^−^, characteristic of pentose derivatives, was much more abundant in isomer with pentose in position 6 than in position 8. Similarly, the [(M−H)−18]^−^ ion was of higher abundance when pentose was in position 6. Thus, peaks f26 and f27 were unambiguously identified as 6-*C*-glucosyl-8-*C*-arabinosyl apigenin (schaftoside) and 6-*C*-arabinosyl-8-*C*-glucosyl apigenin (isoschaftoside) by co-chromatography with corresponding standards (Fig. S1). In general, the sugars involved in the glycosylated flavonoids usually refer to hexose (glucose, galactose, and rhamnose) and pentose (arabinose and xylose) [19]. Accordingly, compounds f24 and f25 were tentatively assigned as 6-*C*-glucosyl-8-*C*-xylosyl apigenin (Ap-6-*C*-Glc-8-*C*-Xyl) and 6-*C-*xylosyl-8-*C*-glucosyl apigenin (Ap-6-*C*-Xyl-8-*C*-Glc) since glucose is the most commonly encountered sugar among glycosylated flavonoids [26]. In a similar way, isomers f22 and f23 were assigned as 6-*C*-Glucosyl-8-*C*-pentosyl luteolin (Lu-6-*C*-Glc-8-*C*-Pen) and 6-*C*-pentosyl-8-*C*-Glucosyl luteolin (Lu-6-*C*-Pen-8-*C*-Glc) considering the UV-vis spectrum and the characteristic fragments [(M−H)−60]^−^ and [(M−H)−18]^−^. Unfortunately, it was difficult to distinguish between xylose and arabinose.

The MS data of isomers f29 and f31 showed there were two substituents: one molecule of glucose and one rhamnose linked to the aglycone apigenin (Table 2). The examples of components f26 and f27 demonstrated that 6-*C*-hexosyl isomers eluted earlier than the 8-*C*-hexosyl with the analysis method in this paper. Hence, peaks f29 and f31 were characterized as 6-*C*-glucosyl-8-*C*-rhamnosyl apigenin (Ap-6-*C*-Glc-8-*C*-Rha) and 6-*C*-rhamnosyl-8-*C*-glucosyl apigenin (Ap-6-*C*-Rha-8-*C*-Glc) [27].

**Table 2:** *C*-glycoside flavonoids identified from lotus tissues.

No	Rt (min)^a^	UV λ_max_ (nm)	ESI-NI (*m/z*)	ESI-PI (*m/z*)	Identification^b^	Ref
f2	14.26	269.8, 348.6	447[M−H]^−^, 327[^0,2^X]^−^, 297[^0,1^X]^−^	449[M+H]^+^, 377[M+H−4H_2_O]^+^, 359[^0,3^X]^+^, 329[^0,2^X]^+^, 299[^0,1^X]^+^	Orientin	Standard
f3	14.58	270.3, 349.3	447[M−H]^−^, 327[^0,2^X]^−^, 297[^0,1^X]^−^	449[M+H]^+^, 377[M+H−4H_2_O]^+^, 359[^0,3^X]^+^, 329[^0,2^X]^+^, 299[^0,1^X]^+^	Isoorientin	Standard
f7	17.06	268.4, 336.8	431[M−H]^−^, 311[^0,2^X]^−^, 281[^0,1^X]^−^	455[M+Na]^+^, 361[M+H−4H_2_O]^+^, 343[^0,3^X]^+^, 313[^0,2^X]^+^, 283[^0,1^X]^+^	Vitexin	Standard
f9	18.41	270.3, 336.1	431[M−H]^−^, 311[^0,2^X]^−^, 281[^0,1^X]^−^	455[M+Na]^+^, 361[M+H−4H_2_O]^+^, 343[^0,3^X]^+^, 313[^0,2^X]^+^, 283[^0,1^X]^+^	Isovitexin	Standard
f21	19.44	272.2, 335.4	593[M−H]^−^, 503[(M−H)−90]^−^, 473[(M−H)−120]^−^, 383[A+113]^−^, 353[A+83]^−^	617[(M+Na]^+^,577[M−H−H_2_O]^+^, 475[(M−H)−120]^+^, 457[^0,2^X−H_2_O]^+^	Ap-6,8-di-*C-*Glc	Ferreres et al., 2003
f22	23.01	270.3, 350.1	579[M−H]^−^, 519[(M−H)−60]^−^, 489[(M−H)−90]^−^, 459[(M−H)−120]^−^, 429[(M−H)−150]^−^, 399[A+113]^−^, 369[A+83]^−^	603[M+Na]^+^, 461[(M−H)−120]^+^, 443[^0,2^X−H_2_O]^+^	Lu-6-*C-*Glc-8-*C-*Pen	
f23	23.66	272.2, 352.0	579[M−H]^−^, 519[(M−H)−60]^−^, 489[(M−H)−90]^−^, 459[(M−H)−120]^−^, 429[(M−H)−150]^−^, 399[A+113]^−^, 369[A+83]^−^	603[M+Na]^+^, 491[(M−H)−90]^+^, 461[(M−H)−120]^+^, 443[^0,2^X−H_2_O]^+^	Lu-6-*C-*Pen-8-*C-*Glc	
f24	26.68	271.7, 335.9	563[M−H]^−^, 503[(M−H)−60]^−^, 473[(M−H)−90]^−^, 443[(M−H)−120]^−^, 383[A+113]^−^, 353[[A+83]^−^	587[M+Na]^+^, 475[(M−H)−90]^+^, 445[(M−H)−120]^+^, 427[^0,2^X−H_2_O]^+^	Ap-6-*C-*Glc-8-*C-*Xyl	Vukics, 2009
f25	28.42	272.2, 335.3	563[M−H]^−^, 545[(M−H)−H_2_O]^−^, 503[(M−H)−60]^−^, 473[(M−H)−90]^−^, 443[(M−H)−120]^−^, 383[A+113]^−^, 353[[A+83]^−^	587[M+Na]^+^, 475[(M−H)−90]^+^, 445[(M−H)−120]^+^, 427[^0,2^X−H_2_O]^+^	Ap-6-*C-*Xyl-8-*C-*Glc	Vukics, 2009
f26	30.13	272.2, 336.7	563[M−H]^−^, 503[(M−H)−60]^−^, 473[(M−H)−90]^−^, 443[(M−H)−120]^−^, 383[A+113]^−^, 353[[A+83]^−^	587[M+Na]^+^, 475[(M−H)−90]^+^, 445[(M−H)−120]^+^, 427[^0,2^X−H_2_O]^+^	Schaftoside	Standard
f27	36.78	271.7, 335.9	563[M−H]^−^, 545[(M−H)−H_2_O]^−^, 503[(M−H)−60]^−^, 473[(M−H)−90]^−^, 443[(M−H)−120]^−^, 383[A+113]^−^, 353[[A+83]^−^	587[M+Na]^+^, 475[(M−H)−90]^+^, 445[(M−H)−120]^+^, 427[^0,2^X−H_2_O]^+^	Isoschaftoside	Standard
f29	43.33	271.9, 334.3	577[M−H]^−^, 503[(M−H)−60]^−^, 457[(M−H)−120]^−^, 383[A+113]^−^, 353[[A+83]^−^	601[M+Na]^+^, 489[(M−H)−90]^+^, 459[(M−H)−120]^+^, 441[^0,2^X−H_2_O]^+^	Ap-6-*C-*Glc-8-*C-*Rha	Vukics, 2009
f31	46.18	272.0, 336.0	577[M−H]^−^, 457[(M−H)−120]^−^, 383[A+113]^−^, 353[[A+83]^−^	601[M+Na]^+^, 489[(M−H)−90]^+^, 459[(M−H)−120]^+^, 441[^0,2^X−H_2_O]^+^	Ap-6-*C-*Rha-8-*C-*Glc	Vukics, 2009

a Rt: retention time on HPLC analysis.

b Lu: luteolin; Ap: Apigenin; Glc: glucose; Pen: pentose; Xyl: xylose; Ara: arabinose; Rha: rhamnose.

### 3. Flavonoid contents

#### 3.1 Flavonoids among different tissues

**Figure 4:**
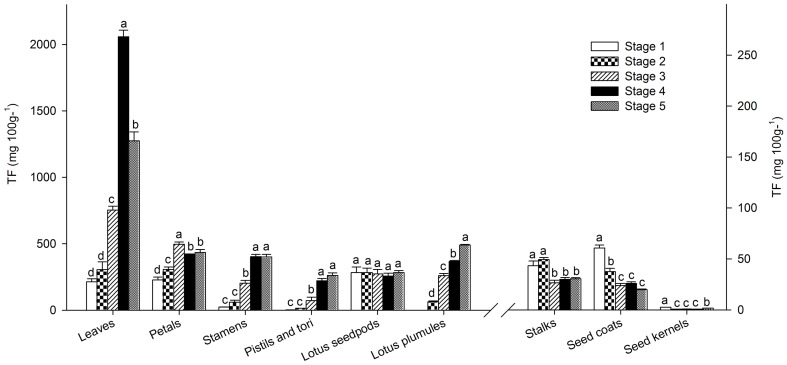
Total flavonoids (TF) during development in various tissues (mean ± SE, n = 3). Bars with no letters in common are significantly different (p<0.05) and the developing stages were corresponded to Fig. S3. Total flavonoids of the first six tissues (leaves, petals, stamens, pistils and tori, lotus seedpods and plumules) used the left ordinate while the remaining three tissues (flower stalks, seed coats and kernels) used the right ordinate.

Flavonoids in each sample were quantified semi-quantitatively by linear regression of rutin at 350 nm and Cy-3-Glc at 525 nm (Table S4, S5). The composition and content of flavonoids varied widely among tissues (Fig. 1, 3, 4). The total flavonoid content (TF), which was defined as the sum total of all the flavonoids measured in this study, was significantly higher in leaves [(2.06±0.08)E3 mg 100 g^−1^, FW] than other tissues (Table S4). Subsequently, flower petals, stamens and lotus plumules exhibited relatively high TF, varying from 402.9±0.30 to 496.0±0.31 mg 100 g^−1^, while the lowest concentrations, 0.67 mg 100 g^−1^, appeared in the seed kernels (Fig. 4). In addition, flavonoids in the seed coats and flower stalks were relatively low as well. The results were in agreement with reported data in various lotus tissues [11].

Corresponding to previous studies, quercetin derivatives were the dominant compounds (≥65.74%) in almost all the tissues, while flower petals and stamens possessed the highest amounts of kaempferol glycosides [11], [14], [28]. Specifically, Qu-3-GlcA (f11) and Ka-3-GlcA (f17) were the prevailing compounds within two derivative groups (Fig. 1, S2). Additionally f14 (Ka-3-Rob) and f16 (Ka-3-Glc) were the predominant compounds in petals. It was reported that Qu-3-GlcA was partly responsible for the elevation of antioxidant activity and that quercetin metabolites could also contribute to antioxidant activity [29]. Lotus leaves produced up to 1.83E3±0.07 mg 100 g^−1^ of Qu-3-GlcA. In addition, Weishan Lake produced appropriately ten thousand kilograms of dessicated lotus leaves each year demonstrating it as a potential source of lotus for the separation and purification of Qu-3-GlcA.

Most importantly, flavonoids in lotus plumules differed significantly from the remaining tissues. Thirteen *C-*glycosyl flavones (newly isolated *C-*glycosides, f7, 9, 21, 22, 23, 24, 25, 26, 27, 29 and 31) and six *O-*glycosides (newly isolated *O-*glycosides, f20, 28, 30, 32 and 30) within plumules were detected. Among them, eleven *C-*glycosides and four *O-*glycosyl flavonoids were reported for the first time in lotus tissues (Table 1, 2). The detection of plentiful *C*-glycosides in lotus might have taxonomic implications.

#### 3.2 Flavonoids among developing stages

Depending on the specific tissue, the TF content increased (leaves, petals, stamens, pistils and tori, and plumules), decreased (flower stalks, seed coats and kernels) or remained constant (lotus seedpods) as the tissues developed (Fig. 4). Qu-3-GlcA was the major flavonoid component contributing to the notably higher TF value observed in leaves, pistils and tori than in other tissues. TF content of leaves reached a maximum at stage 4, while the level was continuously increasing in pistils and tori. In contrast, Qu-3-GlcA led to the decrease of TF within flower stalks, seed coats and kernels. It was noteworthy that TF in seed kernels, although related to plumules, had a slightly increase due to the presence of isovitexin in later stages. The content changes in petals were relatively more complicated than in the tissues mentioned above. The content of Qu-3-GlcA reduced, while levels of Ka-3-Glc and Ka-3-GlcA increased as the petals matured. A relatively greater amount of both Ka-3-Glc and Ka-3-GlcA ultimately increased TF, achieving the highest amount at stage 3 (Fig. 4). Similarly, kaempferol glycosides, especially the compound Ka-3-GlcA, kept pace with the sustaining increase in TF content in flower stamens. The TF value showed that flavonoids in lotus seedpods remained constant during all five stages (Fig. 4). The composition of flavonoids in plumules, which were rich in *C*-flavonoids, was extremely different from other tissues. And TF content increased continuously in accordance with the *C*-glycosyl apigenin derivatives. It was no surprise that flavonoid *C*-glycosides have been receiving considerable attention due to their wide range of biological activities, including anti-inflammatory, antimicrobial, antioxidant, antinociceptive, sedative, antihepatotoxic, antidiabetic, antihypertensive and radioprotective properties [30], [31]. The medical and nutritive applications of lotus warrant further exploration.

### 4. Putative flavonoid biosynthesis pathway of lotus

**Figure 5:**
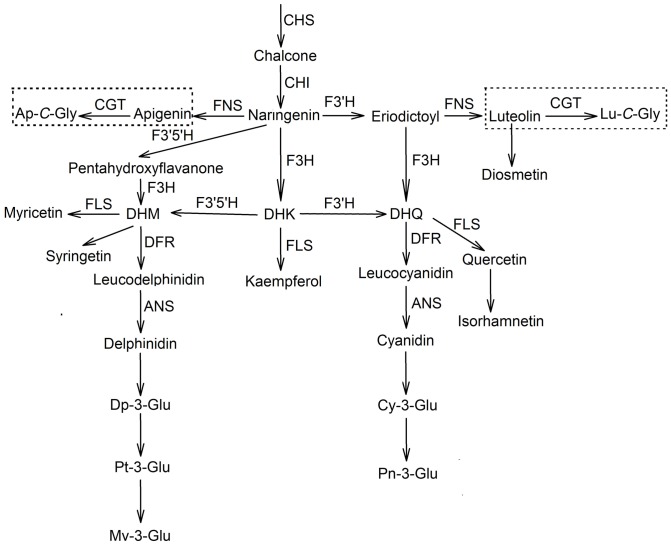
Putative flavonoid biosynthesis pathway related to *N. nucifera*. The biosynthesis pathway within two dotted boxes indicated the *C*-glycosyl flavones in lotus tissues. Ap: apigenin; Lu: luteolin; Dp: delphinidin; Pt: petunidin; Mv: malvidin; Cy: cyanidin; Pn: peonidin; Glc: glocoside; Gly: glycoside; Qu: quercetin; CHS: chalcone synthase; CHI: chalcone isomerase, F3H: flavonoid 3-hydroxylase; F3′H: flavonoid 3′-hydroxylase; F3′5′H: flavonoid 3′,5′-hydroxylase; FNS: flavone synthase; DFR: dihydroflavonol reductase; FLS: flavonol synthase; ANS: anthocyanidin synthase; CGT: *C*- flavonoid glycosyltransferase.

Taking into consideration previous studies and these compounds newly detected in lotus tissues, we propose the putative flavonoid biosynthesis pathway in *N. nucifera* (Fig. 5) [32], [33]. It begins from coumaroyl-CoA and malonyl-CoA. Centered on naringenin, the pathway is divided into nine sub-pathways for synthesis of anthocyanins and flavonoids, with the enzymes of F3′H, FNS, F3H, FLS and F3′5′H. Thereinto, seven sub-pathways synthesize different aglycones of flavonols and flavones (diosmetin, luteolin, quercetin/isorhamnetin, kaempferol, apigenin, myricetin and syringetin), while the remaining two sub-pathways synthesize anthocyanins (delphinidin/petunidin/malvidin, and cyanidin/peonidin). This completes the first important modification of hydroxylation of flavonoids. Subsequently, the resulting secondary metabolites are glycosylated by glycosyltransferase at different positions, resulting in various flavonoid glycosides. Among them, *O*-glycosyl flavonoids are most prevalent in almost all the tissues, whereas *C*-glycosyl flavonoids appear in lotus plumules in large amounts. *C*-flavonoid glycosyltransferase (CGT) is firstly observed in lotus. Considering that CGT is significantly active only in lotus plumules, it may indicate the specific medicinal value of lotus embryos distinctly from its other tissues. However, the exact flavonoid biosynthesis pathway in lotus tissues still needs to be confirmed by further molecular and biochemical evidence.

## Conclusions

In this study, we proposed two sensitive, reliable and reproducible solvent systems that could separate *C*-glycosyl flavonoids in lotus plumules, plus anthocyanins and other flavonoids from the remaining tissues. Flavonoids in nine tissues were studied, and we found that lotus leaves possessed the highest amount of flavonoids, followed by flower petals, lotus plumules and stamens. Moreover, we determined the optimum harvest time for vegetable, tea or medicinal purposes. Overall, thirty-three flavonoids were identified, in which eleven *C*-glycosides and five *O*-glycosides were detected for the first time in lotus tissues. The detection of plentiful *C*-glycosyl flavonoids has enhanced our understanding of flavonoid biosynthesis in lotus. These findings demonstrate the importance of further study of flavonoid *C*-glycosides because of their wide range of biological activities that could prove vital in the use of lotus plumules for medical and nutritional applications.

## Experimental

### 1. Standards and solvents

Cyanidin 3-*O*-β-D-glucopyranoside (a2), luteolin 8-*C*-β-D-glucopyranoside (f2), luteolin 6-*C*-β-D-glucopyranoside (f3), apigenin 8-*C*-β-D-glucopyranoside (f7), apigenin 6-*C*-β-D-glucopyranoside (f9), quercetin 3-*O*-β-D-glucopyranoside (f12) and five aglycone standards (myricetin, apigenin, kaempferol, isorhamnetin, diosmetin) were purchased from Shanghai Tauto Biotech (Shanghai, China). Quercetin 3-*O*-α-L-rhamnopyranosyl-(1→6)-β-D-glucopyranoside (f8) and two aglycone standards (luteolin, quercetin) were obtained from the National Institute for the Control of Pharmaceutical and Biological Products (Beijing, China). Quercetin 3-*O*-β-D-galactopyranoside (f10) was obtained from Chromadex (Laguna Hills, CA, USA). Apigenin 6-*C*-glucoside-8-*C*-arabinoside (f26) and Apigenin 6-*C*-arabinoside-8-*C*-glucoside (f27) were purchased from Beijing Bio-function Biotech (Beijing, China). Kaempferol 3-*O*-β-D-glucopyranoside (f16) and isorhamnetin 3-*O*-β-D-glucopyranoside (f18) were generously offered by Professor Xiao Wang (Shandong Analysis and Test Center, Shandong Academy of Sciences, Shandong, China).

Methanol and acetonitrile used for high-performance liquid chromatography-photodiode array detection/electrospray ionization multistage mass spectrometry (HPLC-DAD/ESI-MS^n^) analysis were of chromatographic grade and purchased from Alltech Scientific (Beijing, China). Trifluoroacetic acid (TFA, ≥99%) was obtained from Merck (Darmstadt, Germany). Other analytical grade chemicals (methanol and formic acid) were obtained from Beijing Chemical Works (Beijing, China). HPLC-grade water was obtained from a Milli-Q System (Millipore, Billerica, MA, USA).

### 2. Plant materials

Nine different tissues including leaves, flower petals, flower stamens, flower pistils and tori, flower stalks, lotus seedpods, seed coats, seed kernels and lotus plumules of *N. nucifera* were collected at Weishan Lake (35°10′N, 116°67′E) in mid-July, 2010 (the authority responsible for the Weishan Lake Wetland Park). No specific permissions were required for these locations/activities, and this study did not involve endangered or protected species. Each tissue was divided into five developmental stages except for lotus plumules since the lotus seed of stage 1 was too unripe to be separated from the seed kernels. There were three divisions of developmental phases: flowers (petals/stamens/pistils and tori/stalks), fruit (seed pods/seed coats/kernels/plumules), and leaves. Each phase was replicated three times using three individual plants (Fig. S3). These materials were first kept overnight in water, then were powdered in liquid nitrogen with mortars and pestles and subsequently stored at −40°C for latter analysis. All concentrations used in this study were calculated from fresh weight.

### 3. Preparation of standard solutions and flavonoid extractions

Standards of Cy-3-Glc and rutin were accurately weighted, separately dissolved in 0.1% HCl-MeOH and MeOH and finally diluted to appropriate concentrations to establish calibration curves at 525 and 350 nm. The others were prepared with MeOH for co-elution with samples and acid hydrolysed solutions.

The extraction method of flavonoids was modified from that of Chen et al [28]. Appropriate amounts of most materials were extracted with methanol-water (70∶30, v/v), while petals were extracted with 70% methanol aqueous solutions containing 0.1% formic acid, shaken in a QL-861 vortex (Kylinbell Lab Instruments, Jiangsu, China), sonicated in KQ-500DE ultrasonic cleaner (Ultrasonic instruments, Jiangsu, China) at 20°C for 20 min, centrifuged in SIGMA 3K30 (Sigma Centrifuges, Germany) (12000 rpm, 10 min), and the supernatant was collected. All extract was further passed through 0.22 µm reinforced nylon membrane filters (Shanghai ANPEL, Shanghai, China) before the HPLC-DAD and HPLC-MS^n^ analyses. Three replicates were performed for each sample.

### 4. Acid hydrolysis of flavonoids extraction

The filtered extract solutions of petals, seed coats and plumules including all the flavonoid components were dried in a rotary evaporator (35°C), re-dissolved in 4 mL 1.5 M HCl in a methanol-water solution (50∶50, v/v) and then heated in a capped tube at 90°C for 2 h. The hydrolysate obtained was partially purified on the Oasis HLB Cartridge (Milford, MA, USA) before HPLC-DAD and HPLC-MS^n^ analysis.

### 5. HPLC-DAD Systems and Conditions

The chromatographic separation was performed on a Dionex (Sunnyvale, CA, USA) system including a P680 HPLC pump, an UltiMate 3000 autosampler, a TCC-100 thermostatted column compartment and a PDA100 photodiode array detector. An aliquot of 10 µL solution was injected and analyzed on an ODS-80Ts QA C18 column (250 mm×4.6 mm, Tosoh, Tokyo, Japan), which was protected with a C18 guard cartridge (Shanghai ANPEL Scientific Instrument, Shanghai, China). Chromatograms were acquired at 525 and 350 nm for anthocyanins and the other flavonoids, respectively, and photodiode array spectra were recorded from 200 to 800 nm. The mobile phase system for all tissues except for plumules was established based on the solution system in blueberry with little modification [34]. Finally, eluent A was 0.1% TFA aqueous solution; eluent B was 15% methanol in acetonitrile (solution system I, S I). A gradient elution as follows was used: 16% B at 0 min, 23% B at 10 min, 26% B at 30 min. The flow rate was 0.8 mL min^−1^.

The separation of flavonoids from lotus plumules was accomplished using the following solvent and gradient: A, 10% formic acid in water; B, formic acid-acetonitrile-water (10∶40∶50, v/v/v); constant gradient from 15 to 45% B within 75 min, at a flow rate of 1.0 mL min^−1^ (solution system II, S II). Unfortunately, two peaks were difficult to achieve in simultaneous separation with the others. Another gradient elution was obtained with a linear elution gradient protocol of 0 min, 15% B; 55 min, 45% B, at a flow rate of 0.8 mL min^−1^. Column temperature was maintained at 35°C for all analyses.

### 6. HPLC-MS^n^ System and Conditions

HPLC-ESI-MS^n^ analysis for anthocyanins and other flavonoids were carried out in an Agilent-1100 HPLC system coupled with a DAD system and a LC-MSD Trap VL electrospray ion mass spectrometer (Agilent Technologies, Palo Alto, CA, USA). The HPLC separation conditions were the same as mentioned above. Anthocyanins were adopted in PI mode and other flavonoids were employed both in PI and in NI mode. The MS conditions were as follows: capillary voltage, 4.0 kV; a nebulization pressure, 241.3 kPa; and a gas (N_2_) temperature, 350°C; flow rate, 8.0 L min^−1^. Capillary offset and exit voltage were separately 74.7 V and 113.0 V for both PI and NI mode. MS spectrum was recorded over the range from *m/z* 50 to 1000.

Since flavonoid *C-*glycosides in lotus plumules needed higher collision energies to fragment than *O-*glycosides, the MS condition was almost the same as the method above with a little modification. Capillary offset and exit voltage were improved to 89.2 V and 151.8 V in PI and NI mode, respectively.

### 7. Statistical Analysis

One-way analysis of variance test (ANOVA) was performed by SPSS 18.0 (SPSS Inc., Chicago, IL). Post hoc comparisons were accomplished with Duncan's test using the same statistical package. The differences were considered to be significant when p<0.05.

## Supporting Information

Figure S1 **Co-chromatography of the** ***C*** **-glycosides with schaftoside and isoschaftoside standards.**
(TIF)

Figure S2 **Changes of flavonoid derivative groups during development in lotus tissues.**
(TIF)

Figure S3 **The developing phases in lotus tissues.**
(TIF)

Table S1 **Linearity of response for Cy-3-Glc and rutin using the optimized method.**
(DOCX)

Table S2 **Intra- and inter-day precision of flavonoids separated by HPLC.**
(DOCX)

Table S3 **UV-vis absorption maxima and main ESI-MS^n^ peaks of anthocyanins separated from** ***N. nucifera*** **petals.**
(DOCX)

Table S4 **Flavonoid contents of various tissues of** ***N. nucifera*** **(A–G, I, mg 100 g^−1^ FW).**
(DOCX)

Table S5 **Flavonoid contents of lotus plumules (mg 100 g^−1^ FW).**
(DOCX)
