# Supplementary figures and images for: Biogenesis of C-Glycosyl Flavones and Profiling of Flavonoid Glycosides in Lotus (Nelumbo nucifera)
Source: PLoS One. 2014 Oct 3;9(10):e108860. doi: 10.1371/journal.pone.0108860 (PMC4184820; doi:10.1371/journal.pone.0108860)

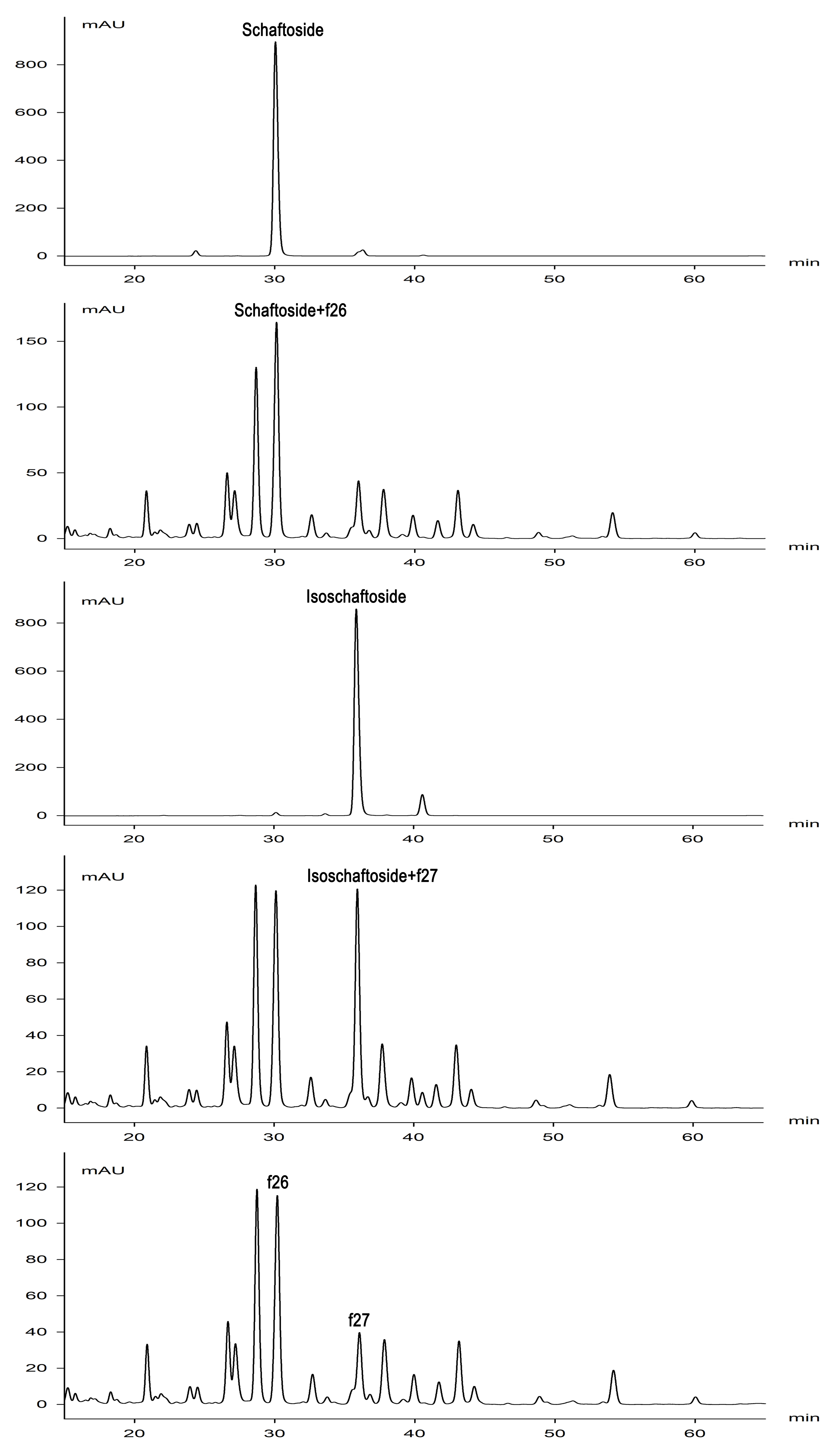

Supplement: Figure S1 — Co-chromatography of the C -glycosides with schaftoside and isoschaftoside standards. (TIF) [file pone.0108860.s001.tif]

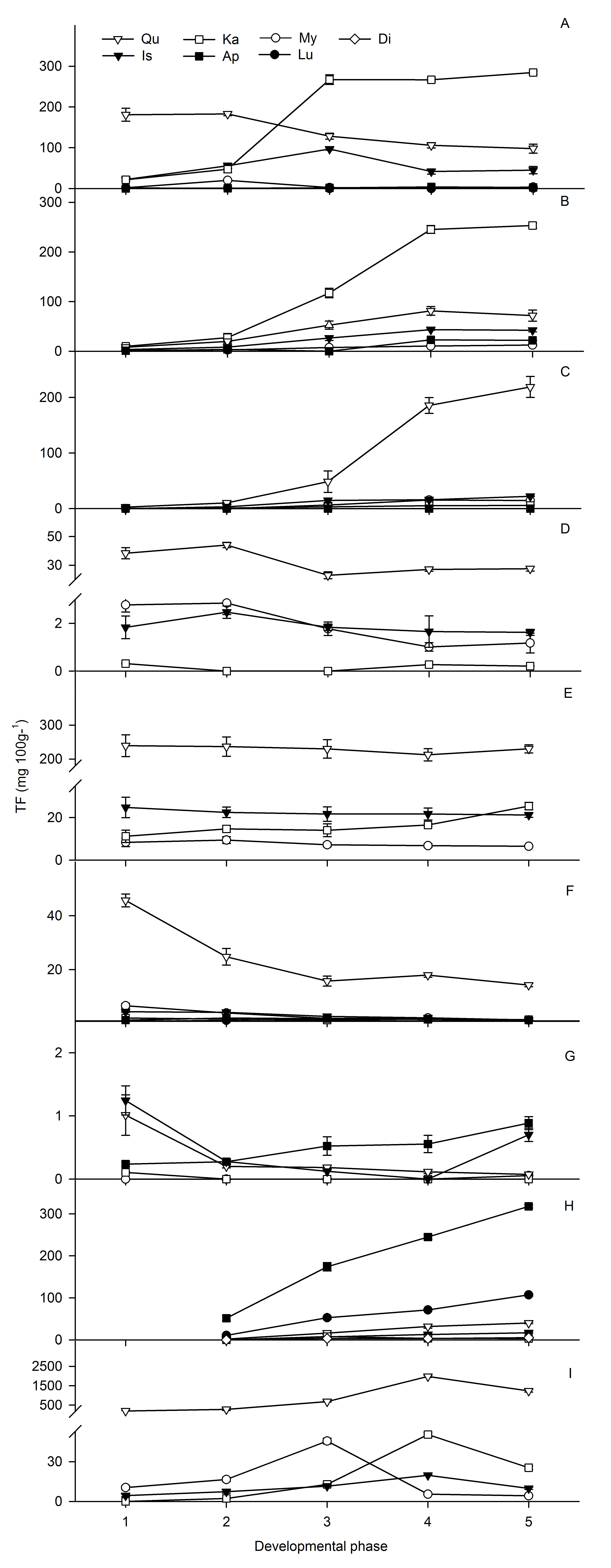

Supplement: Figure S2 — Changes of flavonoid derivative groups during development in lotus tissues. (TIF) [file pone.0108860.s002.tif]

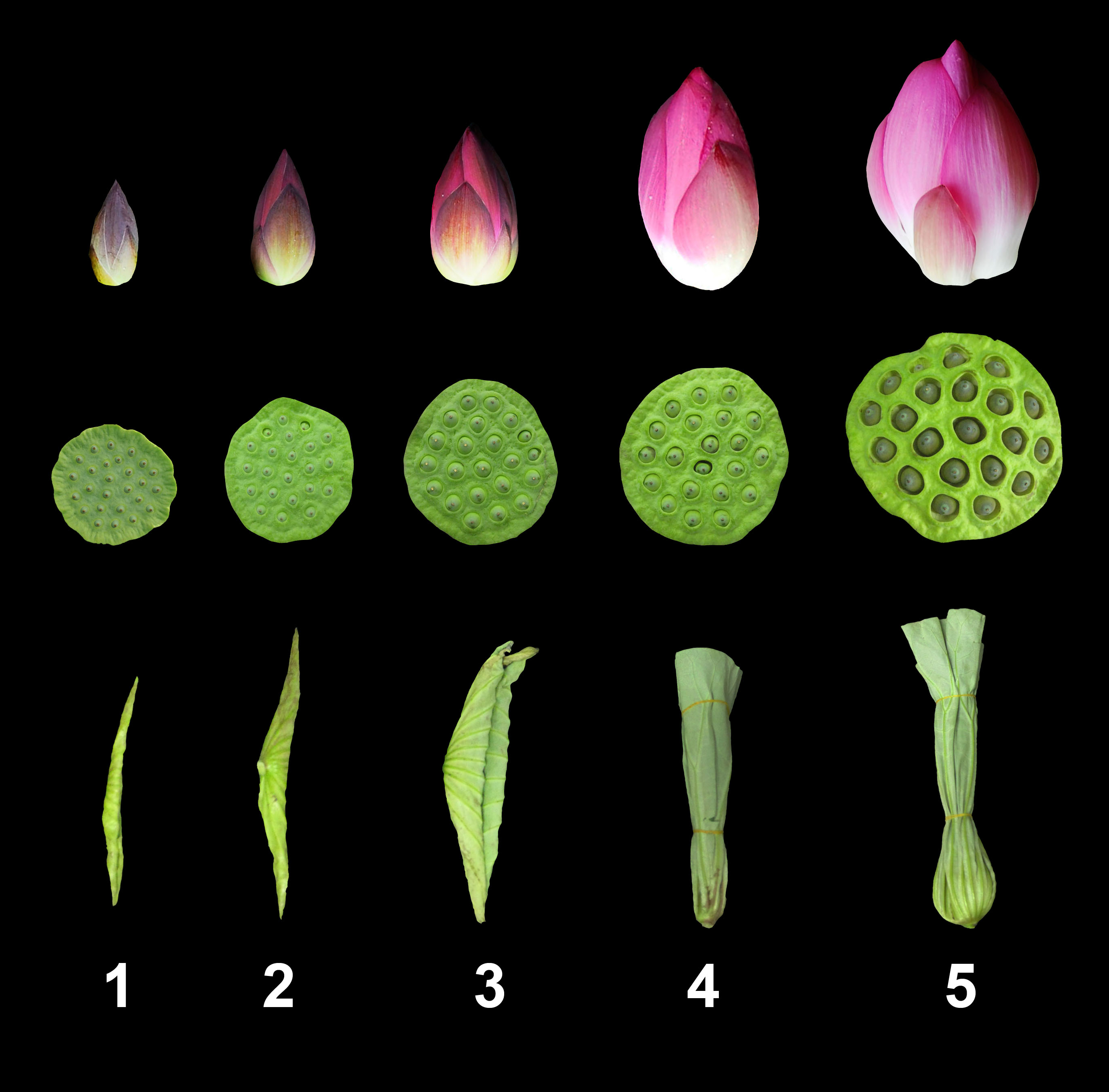

Supplement: Figure S3 — The developing phases in lotus tissues. (TIF) [file pone.0108860.s003.tif]
