# Supplementary material for: Biogenesis of C-Glycosyl Flavones and Profiling of Flavonoid Glycosides in Lotus (Nelumbo nucifera)
Source: PLoS One. 2014 Oct 3;9(10):e108860. doi: 10.1371/journal.pone.0108860 (PMC4184820; doi:10.1371/journal.pone.0108860)
Supplement: Table S1 — Linearity of response for Cy-3-Glc and rutin using the optimized method. (DOCX) [file pone.0108860.s004.docx]

**Table S1.** Linearity of response for Cy-3-Glc and rutin using the optimized method

| Compound | Regression equation^a^ | Regression r^2^ | Linear range (μg mL^-1^) | LOD^b^ (μg mL^-1^) | LOQ^c^ (μg mL^-1^) |
| --- | --- | --- | --- | --- | --- |
| Rutin^d^ | y=350.89x+1.32 | 0.9998 | 12.53−995.48 | 0.28 | 0.94 |
| Rutin^e^ | y= 273.47x + 0.30 | 0.9997 | 15.16−1009.69 | 0.68 | 2.27 |
| Cy-3-Glc | y=474.58x+5.29 | 0.9993 | 8.88−990.27 | 0.51 | 1.68 |

^a^: y, peak area; x, compound concentration(μg mL^-1^);

^b^: LOD=limit of detection, S/N=3;

^c^: LOQ=limit of quantitation, S/N=10;

^d, e^: Calibration curve for standard rutin using analytical method 1 and 2, respectively.
