# Supplementary material for: Biogenesis of C-Glycosyl Flavones and Profiling of Flavonoid Glycosides in Lotus (Nelumbo nucifera)
Source: PLoS One. 2014 Oct 3;9(10):e108860. doi: 10.1371/journal.pone.0108860 (PMC4184820; doi:10.1371/journal.pone.0108860)
Supplement: Table S2 — Intra- and inter-day precision of flavonoids separated by HPLC. (DOCX) [file pone.0108860.s005.docx]

**Table S2.** Intra- and inter-day precision of flavonoids separated by HPLC

| Flavonoids in other tissues | | | | | Flavonoids in lotus plumules | | | | | Anthocyanins in flower petals | | | | |
| --- | --- | --- | --- | --- | --- | --- | --- | --- | --- | --- | --- | --- | --- | --- |
|  | Intra-day (n=6) | | Inter-day (n=3) | |  | Intra-day (n=6) | | Inter-day (n=3) | |  | Intra-day (n=6) | | Inter-day (n=3) | |
| No. ^a^ | Contents (μg mL^-1^) | RSD  (%) | Contents (μg mL^-1^) | RSD ^c^  (%) | No. | Contents  (μg mL^-1^) | RSD  (%) | Contents (μg mL^-1^) | RSD  (%) | No. | Contents (μg mL^-1^) | RSD  (%) | Contents (μg mL^-1^) | RSD  (%) |
| f1 | 4.50±0.04 ^b^ | 2.00 | 4.35±0.001 | 0.06 | f21 | 40.43±0.20 | 1.23 | 40.59±0.10 | 0.44 | a1 | 25.57±0.05 | 0.52 | 26.21±0.31 | 2.06 |
| f2 | 0.84±0.01 | 2.35 | 0.82±0.01 | 2.10 | f22 | 11.38±0.04 | 0.96 | 11.48±0.05 | 0.7 | a2 | 8.79±0.05 | 1.27 | 9.30±0.12 | 2.15 |
| f3 | 1.54±0.001 | 0.26 | 1.58±0.002 | 0.30 | f23 | 10.59±0.03 | 0.74 | 10.60±0.03 | 0.43 | a3 | 21.79±0.06 | 0.73 | 23.17±0.08 | 0.63 |
| f4 | 17.61±0.03 | 0.39 | 17.72±0.02 | 0.16 | f24 | 77.43±0.67 | 2.13 | 77.54±0.11 | 0.24 | a4 | 17.51±0.15 | 2.17 | 19.06±0.10 | 0.95 |
| f5 | 5.22±0.003 | 0.16 | 5.21±0.002 | 0.10 | f2 | 71.48±0.22 | 0.76 | 71.47±0.13 | 0.31 | a5 | 80.24±0.34 | 1.04 | 82.16±0.17 | 0.36 |
| f6 | 9.03±0.01 | 0.22 | 9.02±0.01 | 0.12 | f25 | 164.74±0.42 | 0.63 | 165.98±0.39 | 0.41 |  |  |  |  |  |
| f7 | 1.62±0.001 | 0.22 | 1.62±0.001 | 0.08 | f3 | 60.16±0.14 | 0.56 | 60.31±0.11 | 0.3 |  |  |  |  |  |
| f8 | 33.64±0.24 | 1.72 | 34.24±0.01 | 0.04 | f26 | 130.9±0.48 | 0.89 | 131.35±0.35 | 0.46 |  |  |  |  |  |
| f9 | 2.07±0.001 | 0.21 | 2.15±0.002 | 0.23 | f7 | 28.91±0.15 | 1.24 | 29.82±0.12 | 0.72 |  |  |  |  |  |
| f10 | 3.72±0.002 | 0.14 | 3.75±0.001 | 0.06 | f10 | 17.2±0.08 | 1.18 | 17.24±0.08 | 0.82 |  |  |  |  |  |
| f11 | 200.47±0.96 | 1.17 | 203.15±0.11 | 0.09 | f27 | 59.64±0.27 | 1.10 | 59.98±0.28 | 0.81 |  |  |  |  |  |
| f12 | 5.16±0.003 | 0.17 | 6.21±0.004 | 0.13 | f28 | 70.94±0.38 | 1.30 | 71.67±0.32 | 0.76 |  |  |  |  |  |
| f13 | 0.88±0.002 | 0.72 | 0.96±0.001 | 0.32 | f9 | 32.42±0.18 | 1.33 | 32.49±0.02 | 0.13 |  |  |  |  |  |
| f14 | 88.55±0.34 | 0.95 | 88.71±0.91 | 1.77 | f29 | 19.92±0.04 | 0.54 | 19.91±0.20 | 1.75 |  |  |  |  |  |
| f15 | 60.07±0.22 | 0.88 | 60.41±0.01 | 0.04 | f30 | 57.54±0.05 | 0.21 | 57.51±0.83 | 2.51 |  |  |  |  |  |
| f16 | 99.21±0.39 | 0.95 | 100.17±0.13 | 0.23 | f31 | 18.42±0.03 | 0.38 | 15.72±0.02 | 0.27 |  |  |  |  |  |
| f17 | 81.29±0.27 | 0.82 | 81.06±0.16 | 0.34 | f14 | 5.98±0.05 | 2.11 | 6.02±0.04 | 1.15 |  |  |  |  |  |
| f18 | 44.11±0.17 | 0.96 | 44.84±0.10 | 0.40 | f32 | 35.45±0.21 | 1.47 | 35.91±0.03 | 0.14 |  |  |  |  |  |
| f19 | 1.37±0.01 | 1.45 | 1.40±0.003 | 0.43 | f33 | 6.45±0.04 | 1.48 | 6.56±0.002 | 0.07 |  |  |  |  |  |
| f20 | 8.15±0.04 | 1.15 | 8.11±0.09 | 1.85 |  |  |  |  |  |  |  |  |  |  |

^a^: The numbers assigned to compounds in accord with those used in Fig. 1, 2;

^b^: Mean content ± SE;

^c^: RSD=(SD/mean)×100.
