# Supplementary material for: Biogenesis of C-Glycosyl Flavones and Profiling of Flavonoid Glycosides in Lotus (Nelumbo nucifera)
Source: PLoS One. 2014 Oct 3;9(10):e108860. doi: 10.1371/journal.pone.0108860 (PMC4184820; doi:10.1371/journal.pone.0108860)
Supplement: Table S3 — UV-vis absorption maxima and main ESI-MSn peaks of anthocyanins separated from N. nucifera petals. (DOCX) [file pone.0108860.s006.docx]

**Table S3.** UV-vis absorption maxima and main ESI-MS^n^ peaks of anthocyanins separated from *N.* *nucifera* petals

| No. | Rt (min)^a^ | UV λ_max_ (nm) | ESI-PI (m/z) | Identification | Ref |
| --- | --- | --- | --- | --- | --- |
| a1 | 9.90 | 278.2，525.4 | 303[Y_0_]^+^、465[M+H] ^+^ | Delphinidin 3-*O-*glucoside (Dp-3-Glc) | Yang et al., 2009 |
| a2 | 11.98 | 280.8，517.6 | 287[Y_0_] ^+^、449[M+H] ^+^ | Cyanidin 3-*O-*glucoside (Cy-3-Glc) | Yang et al., 2009 |
| a3 | 12.92 | 275.9，527.1 | 317[Y_0_] ^+^、479[M+H] ^+^ | Petunidin 3-*O-*glucoside (Pt-3-Glc) | Yang et al., 2009 |
| a4 | 15.25 | 279.8，519.2 | 301[Y_0_] ^+^、463[M+H] ^+^ | Peonidin 3-*O-*glucoside (Pn-3-Glc) | Yang et al., 2009 |
| a5 | 16.02 | 278.5，528.4 | 331[Y_0_] ^+^、493[M+H] ^+^ | Malvidin 3-*O-*glucoside (Mv-3-Glc) | Yang et al., 2009 |

^a^ Rt: retention time on HPLC analysis.
