# Supplementary material for: Biogenesis of C-Glycosyl Flavones and Profiling of Flavonoid Glycosides in Lotus (Nelumbo nucifera)
Source: PLoS One. 2014 Oct 3;9(10):e108860. doi: 10.1371/journal.pone.0108860 (PMC4184820; doi:10.1371/journal.pone.0108860)
Supplement: Table S4 — Flavonoid contents of various tissues of N. nucifera (A–G, I, mg 100 g−1 FW). (DOCX) [file pone.0108860.s007.docx]

**Table S4.** Flavonoid contents of various tissues of *N. nucifera* (A-G, I, mg 100g^-1^ FW)

|  | f1 | f2 | f3 | f4 | f5 | f6 | f7 | f8 | f9 | f10 | f11 | f12 | f13 | f14 | f15 | f16 | f17 | f18 | f19 | f20 |
| --- | --- | --- | --- | --- | --- | --- | --- | --- | --- | --- | --- | --- | --- | --- | --- | --- | --- | --- | --- | --- |
| A-1 | 0.82±0.33 | 3.64±0.55 | - | - | - | - | 0.83±0.10 | 4.15±1.84 | 1.65±0.00 | 1.44±0.10 | 90.99±9.04 | 1.26±0.00 | - | 21.30±0.65 | 13.54±6.32 | 132.54±1.81 | 130.67±5.15 | 24.82±1.49 | 6.66±1.57 | - |
| A-2 | 0.89±0.30 | - | - | - | - | - | 0.95±0.16 | 6.27±2.88 | 2.76±0.00 | 1.55±0.09 | 98.20±6.11 | - | - | 26.97±1.47 | 12.15±3.02 | 135.57±4.90 | 104.01±3.44 | 24.90±4.39 | 4.67±1.44 | - |
| A-3 | 2.26±0.78 | - | - | - | - | - | 1.57±0.27 | 6.46±0.33 | - | 1.98±0.40 | 118.63±6.88 | 1.27±0.00 | - | 35.04±0.90 | 35.20±7.82 | 101.87±4.94 | 130.11±6.22 | 53.47±9.23 | 8.16±0.97 | - |
| A-4 | 1.19±0.03 | 0.88±0.10 | - | 14.54±0.43 | 4.25±0.03 | 8.60±0.83 | - | 5.87±0.12 | 0.68±0.01 | 2.13±0.33 | 166.18±4.99 | - | - | 7.51±1.72 | 19.17±0.84 | 23.60±7.19 | 16.29±0.30 | 32.91±5.82 | 3.55±0.13 | 2.93±0.52 |
| A-5 | 1.76±0.55 | 0.64±0.11 | 0.46±0.01 | - | - | - | - | 7.21±0.79 | - | 2.11±0.42 | 171.54±15.87 | - | - | 3.68±0.22 | 9.64±0.61 | 10.21±1.97 | 7.53±1.82 | 12.45±3.76 | - | 4.20±0.01 |
| B-1 | - | - | - | 12.33±1.45 | - | 4.28±0.55 | - | 15.82±0.83 | 21.77±0.49 | 1.78±0.42 | 49.13±10.27 | 0.68±0.00 | - | 12.57±1.2 | 7.99±2.22 | 51.82±2.16 | 188.77±4.45 | 29.19±2.51 | 5.05±2.13 | - |
| B-2 | - | - | - | 10.36±0.70 | - | 4.15±0.62 | - | 15.96±0.53 | 22.88±0.12 | 1.61±0.29 | 59.52±9.18 | - | - | 13.46±1.80 | 9.79±3.47 | 66.96±3.14 | 164.75±6.15 | 27.77±3.14 | 5.74±0.68 | - |
| B-3 | - | - | - | 7.34±1.56 | - | 3.86±1.77 | - | 9.44±0.35 | - | 0.78±0.08 | 37.56±6.21 | 0.84±0.01 | - | 7.57±0.48 | 4.96±2.55 | 22.44±0.41 | 87.06±9.10 | 16.76±2.37 | 4.73±0.20 | - |
| B-4 | - | - | - | 2.53±0.64 | - | 1.86±0.26 | - | 5.65±0.55 | 3.47±0.99 | - | 12.20±3.28 | - | - | 3.63±2.25 | 1.43±0.43 | 6.03±2.07 | 17.51±5.12 | 4.42±1.93 | 2.23±0.10 | - |
| B-5 | - | - | - | 1.88±0.33 | - | 0.25±0.00 | - | 1.08±0.04 | 0.93±0.17 | - | 6.55±0.39 | - | - | 0.79±0.03 | 0.70±0.04 | 2.61±0.14 | 6.37±0.22 | 1.89±0.07 | 0.75±0.01 | - |
| C-1 | 1.04±0.41 | - | - | 13.35±0.68 | - | 5.48±2.79 | - | 2.10±0.54 | - | 1.06±0.13 | 208.92±16.71 | 1.32±0.01 | - | 0.67±0.01 | 3.47±1.35 | 1.93±0.43 | 3.06±0.70 | 13.11±1.69 | 5.52±0.06 | - |
| C-2 | 1.14±0.41 | - | - | 14.27±0.63 | - | 8.45±0.80 | - | 2.65±0.95 | - | 0.83±0.03 | 172.59±14.37 | 0.99±0.00 | - | - | 3.51±0.75 | 2.10±0.30 | 3.06±0.90 | 12.33±2.18 | - | - |
| C-3 | 1.58±0.52 | - | - | 4.73±2.05 | - | 3.48±1.15 | - | 2.28±0.86 | - | - | 42.61±17.50 | - | - | 0.94±0.01 | 4.37±2.07 | 0.83±0.03 | 1.37±0.32 | 6.23±2.36 | 4.03±0.28 | - |
| C-4 | - | - | - | - | - | 1.03±0.23 | - | 1.12±0.07 | - | - | 8.02±0.77 | - | - | - | 0.90±0.08 | - | 0.93±0.10 | 0.91±0.19 | 1.42±0.06 | - |
| C-5 | - | - | - | - | - | - | - | 0.80±0.09 | 0.58±0.03 | - | 1.80±0.21 | - | - | - | - | - | - | - | - | - |
| D-1 | 0.22±0.00 | - | - | 0.96±0.42 | - | 0.94±0.47 | - | 0.40±0.17 | - | 0.55±0.01 | 25.67±1.31 | - | - | - | 0.35±0.10 | - | 0.21±0.00 | 0.59±0.06 | 0.68±0.09 | - |
| D-2 | 0.27±0.00 | - | - | 0.74±0.18 | - | 0.86±0.11 | - | 0.40±0.05 | - | 0.39±0.05 | 25.06±1.35 | 0.37±0.01 | - | - | 0.31±0.03 | - | 0.27±0.00 | 0.65±0.32 | 0.70±0.33 | - |
| D-3 | 0.33±0.14 | - | - | 1.44±0.15 | - | 0.61±0.07 | - | 0.41±0.13 | - | 0.23±0.02 | 21.70±2.38 | - | - | - | 0.34±0.09 | - | - | 0.62±0.08 | 0.88±0.05 | - |
| D-4 | 0.54±0.22 | - | - | 2.31±0.41 | - | 1.15±0.39 | - | 1.35±0.68 | - | 0.48±0.09 | 41.13±1.03 | - | - | - | 0.75±0.24 | - | - | 0.86±0.12 | 0.86±0.21 | - |
| D-5 | 0.55±0.22 | - | - | 2.22±0.50 | - | 2.12±0.84 | - | 1.14±0.50 | - | 0.41±0.04 | 34.73±4.60 | - | - | - | 0.64±0.03 | - | 0.31±0.02 | 0.54±0.23 | 0.65±0.27 | - |
| E-1 | - | - | - | 8.37±1.98 | - | 6.16±0.34 | - | - | - | 1.19±0.02 | 231.90±31.74 | - | - | 1.57±0.16 | 2.12±0.61 | 3.64±1.45 | 6.00±1.3 | 14.62±3.52 | 8.06±0.90 | - |
| E-2 | 0.85±0.01 | - | - | 8.58±1.59 | - | 3.28±0.81 | - | 3.42±0.39 | - | 0.95±0.04 | 229.01±29.03 | - | - | 1.63±0.14 | 4.03±1.39 | 4.32±0.31 | 8.73±1.54 | 11.10±0.32 | 7.33±1.13 | - |
| E-3 | - | - | - | 7.21±0.93 | - | 3.18±0.42 | - | 1.55±0.42 | - | 1.19±0.03 | 223.92±27.29 | - | - | 1.18±0.15 | 3.03±0.98 | 4.31±0.94 | 8.55±2.22 | 10.66±2.69 | 8.02±1.24 | - |
| E-4 | - | - | - | 6.81±0.86 | - | 3.03±0.36 | - | - | - | 0.61±0.01 | 208.82±17.66 | - | - | 0.83±0.06 | 1.02±0.20 | 4.55±0.40 | 11.15±0.54 | 11.92±2.09 | 8.78±0.56 | - |
| E-5 | - | - | - | 6.50±0.48 | - | 3.73±0.12 | - | 0.94±0.05 | - | 2.19±0.11 | 223.24±11.75 | - | - | 1.32±0.14 | 1.09±0.07 | 8.45±1.39 | 15.67±0.36 | 11.72±1.12 | 8.46±0.15 | - |
| F-1 | - | - | 0.45±0.04 | 6.60±0.86 | - | 0.4±0.02 | 0.58±0.05 | - | 0.66±0.08 | - | 45.30±2.38 | - | - | 0.24±0.02 | 0.80±0.09 | 0.96±0.13 | 0.82±0.18 | 2.61±0.22 | 0.95±0.13 | - |
| F-2 | - | 0.51±0.04 | 0.67±0.05 | 3.92±0.15 | - | 0.26±0.04 | 0.94±0.09 | 0.15±0.00 | 1.06±0.11 | - | 24.35±3.08 | - | - | 0.27±0.04 | 1.14±0.25 | 0.96±0.04 | 0.43±0.10 | 1.36±0.21 | 1.64±0.11 | - |
| F-3 | - | 0.24±0.00 | 0.38±0.03 | 1.87±0.06 | - | 0.26±0.00 | 0.95±0.07 | - | 0.84±0.03 | - | 14.30±1.56 | 1.19±0.35 | - | 0.23±0.03 | 0.73±0.21 | 0.74±0.05 | 0.29±0.06 | 1.01±0.32 | 0.87±0.11 | - |
| F-4 | - | 0.22±0.04 | 0.43±0.06 | 2.04±0.60 | - | 0.61±0.01 | 0.76±0.09 | - | 0.74±0.07 | - | 15.99±0.72 | 1.38±0.08 | 0.17±0.02 | 0.18±0.02 | 0.35±0.12 | 0.90±0.10 | 0.41±0.08 | 1.23±0.14 | 0.52±0.17 | - |
| F-5 | 0.29±0.06 | - | 0.34±0.02 | 0.99±0.22 | - | 0.25±0.02 | 0.66±0.08 | - | 0.63±0.07 | - | 13.01±0.36 | 0.99±0.24 | 0.17±0.02 | 0.19±0.00 | 0.23±0.01 | 0.80±0.01 | 0.29±0.03 | 1.04±0.05 | - | - |
| G-1 | - | - | - | - | - | 0.36±0.27 | - | - | 0.24±0.04 | - | 0.43±0.03 | 0.22±0.04 | - | - | 0.50±0.00 | 0.11±0.02 | - | 0.27±0.00 | 0.47±0.24 | - |
| G-2 | - | - | - | - | - | - | - | - | 0.27±0.02 | - | 0.20±0.02 | - | - | - | - | - | - | 0.07±0.00 | 0.21±0.02 | - |
| G-3 | - | - | - | - | - | - | - | 0.10±0.01 | 0.52±0.15 | - | 0.08±0.04 | - | - | - | - | - | - | - | 0.12±0.00 | - |
| G-4 | - | - | - | - | - | - | - | 0.08±0.02 | 0.56±0.14 | - | 0.03±0.03 | - | - | - | - | - | - | - | - | - |
| G-5 | - | - | - | 0.05±0.00 | - | - | - | 0.07±0.01 | 0.89±0.10 | - | - | - | - | - | 0.05±0.01 | - | - | 0.12±0.00 | 0.53±0.11 | - |
| I-1 | 2.88±0.01 | - | - | 7.60±1.54 | - | 10.53±0.72 | - | 4.32±2.23 | - | - | 1.85E2±0.37 | - | - | - | 1.81±0.10 | - | - | 2.59±0.74 | - | - |
| I-2 | 2.63±0.00 | - | - | 6.06±1.51 | 7.87±0.01 | 9.16±0.82 | - | 4.98±2.20 | - | 2.33±0.51 | 2.63E2±0.96 | - | - | - | 3.10±0.44 | - | 2.25±0.01 | 4.27±1.09 | - | - |
| I-3 | - | - | - | 5.61±1.50 | 40.03±0.85 | - | - | 5.65±0.26 | - | 15.84±0.67 | 6.62E2±0.44 | - | 1.87±0.08 | 1.78±0.08 | 1.78±0.07 | 1.90±0.17 | 7.31±0.45 | 4.66±0.07 | 5.07±0.06 | - |
| I-4 | - | - | - | 5.45±0.50 | - | 84.33±1.31 | - | 12.59±0.89 | - | 53.65±1.80 | 1.83E3±0.08 | - | 5.34±0.17 | 3.85±0.18 | 1.38±0.00 | 15.35±0.50 | 26.05±0.90 | 9.71±1.06 | 8.65±0.68 | 16.32±1.01 |
| I-5 | - | - | - | 4.27±0.07 | - | 62.77±3.32 | - | 5.89±0.32 | - | 25.19±1.31 | 1.14E3±0.10 | - | 2.29±0.12 | 1.87±0.09 | - | 6.51±0.31 | 14.83±0.89 | 4.77±0.79 | 5.11±0.37 | - |

^a^ The figure numbers and the developing stages (A-1 to I-5) were accorded with Fig.1, 2, S3. A: flower petals, B: stamens, C: pistils and tori, D: flower stalks, E: lotus seedpods, F: seed coats, G: kernels, H: embryos, I: lotus leaves.
