# Supplementary material for: Biogenesis of C-Glycosyl Flavones and Profiling of Flavonoid Glycosides in Lotus (Nelumbo nucifera)
Source: PLoS One. 2014 Oct 3;9(10):e108860. doi: 10.1371/journal.pone.0108860 (PMC4184820; doi:10.1371/journal.pone.0108860)
Supplement: Table S5 — Flavonoid contents of lotus plumules (mg 100 g−1 FW). (DOCX) [file pone.0108860.s008.docx]

**Table S5.** Flavonoid contents of lotus plumules (mg 100g^-1^ FW)

| No | H-2 | H-3 | H-4 | H-5 |
| --- | --- | --- | --- | --- |
| f21 | 3.26±0.45 | 13.18±0.47 | 18.68±1.40 | 25.03±0.81 |
| f22 | 0.99±0.09 | 5.38±1.53 | 5.66±0.64 | 6.49±0.09 |
| f23 | 0.91±0.05 | 5.15±1.48 | 5.79±0.25 | 6.07±0.18 |
| f24 | 8.42±0.95 | 23.05±1.37 | 34.36±3.73 | 46.03±0.84 |
| f2 | 4.43±0.21 | 15.45±0.35 | 26.82±0.45 | 36.99±1.48 |
| f25 | 17.70±2.05 | 51.99±1.39 | 73.94±6.08 | 90.80±1.77 |
| F3 | 0.96±0.04 | 5.77±0.05 | 7.12±0.88 | 28.07±1.48 |
| f26 | 11.45±1.83 | 44.08±2.99 | 60.27±4.17 | 72.43±1.15 |
| f7 | 1.59±0.16 | 6.72±1.53 | 8.71±1.41 | 14.72±0.29 |
| f10 | - | 3.13±0.19 | 5.44±0.78 | 7.04±0.42 |
| f27 | 4.86±0.48 | 16.85±1.03 | 24.21±2.59 | 32.89±0.66 |
| f28 | 1.77±0.13 | 12.98±0.57 | 26.20±3.02 | 33.06±1.57 |
| f9 | 1.17±0.08 | 6.89±1.55 | 8.48±1.03 | 15.12±0.35 |
| f29 | 1.97±0.24 | 6.31±0.22 | 9.53±0.89 | 11.62±0.43 |
| f30 | 3.20±0.27 | 21.01±0.91 | 25.87±1.68 | 29.38±0.20 |
| f31 | 1.13±0.11 | 4.85±0.47 | 6.64±0.56 | 9.26±0.29 |
| f14 | - | 7.79±0.39 | 3.55±0.80 | 3.23±0.13 |
| f32 | - | 6.97±0.48 | 12.64±1.78 | 16.82±1.41 |
| f33 | - | 3.24±0.49 | 3.15±0.19 | 5.07±1.35 |

^a^ The figure numbers and the developing stages (H-2 to H-5) were accorded with Fig.1, 2, S3.
